# Knowledge of Breastfeeding Practices Among Mothers Attending a Tertiary Care Setting in East India

**DOI:** 10.7759/cureus.37146

**Published:** 2023-04-04

**Authors:** Prasanta Rajak, Anusree Krishna mandal, Jadab Kumar Jana, Soumya Gayen

**Affiliations:** 1 Pediatrics, Bankura Sammilani Medical College and Hospital, Bankura, IND

**Keywords:** neonatal health, exclusive breast feeding (ebf), exclusive breast feeding, awareness, rural, mother, nutrition, breastfeeding

## Abstract

Background

Human milk offers a neonate a balanced diet for healthy growth and development, in addition to its myriad of benefits like preventing stunting, protecting against infectious and chronic diseases, and decreasing infant mortality.

Objective

To assess the knowledge of mothers and other factors that contribute to breastfeeding practices.

Methods

This is a one-year hospital-based cross-sectional study that included 400 mothers who followed up with the hospital for the healthcare of their child, aged between six and 24 months. A survey was used for data collection.

Results

Ninety-three percent of the mothers were from the countryside, and 78% of them were under 25 years of age. Eighty-seven percent of mothers worked at home, while 83% of mothers were part of nuclear households. Ninety-nine percent of mothers delivered their neonates at a medical facility, and 77% of mothers did so for the first time. Only 53% of mothers resorted to exclusive breastfeeding (EBF), even though 68% of mothers were aware of its significance. Thirty-six percent of mothers adopted EBF, while only 23% of women were aware that breastfeeding should be started within the first hour of childbirth. Working women (p=0.000), mothers with several children (p=0.000), mothers older than 25 years of age (p=0.002), and mothers with higher education levels than the 10th grade (p=0.000) showed good understanding and practice of breastfeeding, which was statistically significant (p<0.5).

Conclusion

The levels of breastfeeding awareness and practice among mothers fell short of both national statistics and WHO recommendations. All helpful information about breastfeeding should be shared with the community at large to improve the data currently available.

## Introduction

A neonate's first natural food is human milk, which provides them with all the energy and nutrients they require during their first few months of life. Effective breastfeeding practises reduce newborn and child mortality and morbidity from diarrhoea, otitis media, necrotizing enterocolitis, and sudden infant death syndrome [1]. It is generally known that breastfeeding has a positive effect on pregnancy intervals in mothers by encouraging lactational amenorrhea and decreasing breast and ovarian cancer rates [1, 2]. The best breastfeeding practices include exclusive breastfeeding (EBF) for the first six months of life, early breastfeeding beginning within the first hour of life, and continuing breastfeeding until or past two years of age [1]. In almost all countries, more than 80% of newborns receive human milk, but only about half initiate nursing during the first hour of life, and rates of exclusive breastfeeding are far below 50% in the majority of the nations [3].

India has the highest rate of infant mortality in the world and is responsible for 20% of the 5.9 million child fatalities worldwide [4]. According to United Nations Children's Fund (UNICEF) India Statistics 2015, India's infant and under-five mortality rates are 48 and 38 per 1,000 live births, respectively, and nearly 50% of these deaths are caused by malnutrition [5]. As per UNICEF India figures from 2015, only 65% of infants who are six months old are exclusively breastfed, and only 45% of infants undergo timely initiation of breastfeeding. These numbers are significantly below the suggested norms.

The best breastfeeding techniques depend on a number of variables, including socio-cultural factors, factors related to health services, community and family factors, and characteristics of the mother and the child [6]. In order to assist policymakers with interventional programmes, this study will attempt to assess breastfeeding knowledge among mothers visiting a hospital for their child's illness that is located in a remote area of India, as well as the factors that influence inappropriate breastfeeding practices.

## Materials and methods

Objective

The study aims to assess the knowledge and practises of breastfeeding among women attending a tertiary care facility for the healthcare of their child, situated in a remote location in Bankura, West Bengal, India.

Study area

The study was carried out in Bankura Sammilani Medical College and Hospital's (BSMCH) paediatric outpatient department (OPD). Eastern India is the location of this study, which involved 400 participants and is the largest prospective study so far to be conducted in this region over the previous ten years.

Study design

A hospital-based cross-sectional study was carried out over the period from January 1 to June 30, 2022.

Study population

Inclusion Criteria

Mothers who had at least one living child aged between six months and two years, who were visiting the paediatric outpatient department for their child's illness, and who had consented to participate in the proposed research activity, met the inclusion criteria.

Exclusion Criteria

Women who did not volunteer to participate in the proposed research study, had medical reasons not to breastfeed, or had adopted a child were excluded from the study.

Mothers meeting the inclusion criteria were randomly chosen from the paediatric OPD. After obtaining written consent, four study participants on each working day, four days a week, making a total of sixteen participants per week, were enrolled for a single face-to-face interview. An interview with a research participant took, on average, 15 minutes.

Sample size determination 

Four hundred {calculation of sample size: n=(Zα)2×P × (1-P)/d2, where Zα=standard normal variate that is 1.96 at 95% confidence interval, p=prevalence of interest of event (prevalence of EBF from the previous study is 65%)} [6] and d=error of absolute precision, which is 5%. Putting all the values into the above formula, n= (1.96)2*0.65*0.35/(0.05)2 = 350. Taking 15% as the nonresponsive rate, the final sample size (n) was 400.

Study tools 

A predesigned, pretested questionnaire was administered by the interviewer. The questionnaire, which contained both closed-ended and open-ended questions to fulfil our study objectives, was prepared after pilot testing with 40 mothers. Those who were participating in the said testing had been later excluded from the study. The questionnaire had 30 questions divided into three groups: group A (11 questions related to demographic characteristics), group B (11 questions related to breastfeeding knowledge), and group C (eight questions related to the practice of breastfeeding among study participants). A scaled scoring system had been developed for selected questions on breastfeeding knowledge (11 questions) and practises (eight questions) and categorised into good, fair, and poor. Each correct answer was worth one point. No points were given for wrong or no answers. The scoring system was as follows: knowledge: good (7-11 points), fair (4-6 points), and poor (0-3 points); practise: good (4-8 points), fair (2-3 points), and poor (0-1 point).

Ethical consideration and consent

The Institutional Ethics Committee gave its clearance before data collection (memo number: BSMC/Aca:3625, dated: 07.12.2021).

Data analysis

Analysing data software called Epi Info (version 3.5.1) was used to analyse the data, which was entered into a Microsoft Excel spreadsheet. Ratios and percentages were used to represent categorical data, as well as the mean and standard deviation for continuous data. The chi-square test was used to analyse the relationship between two variables, and a p-value of < 0.05 was accepted as statistically significant.

## Results

Socio-demographic characteristics

Four hundred mothers were interviewed; 78% of them were ≤ 25 years old, and 93% hailed from rural areas. Only 22% and 5% of mothers were younger than 20 and older than 30 years of age, respectively. In terms of education, 67% of mothers had a high school diploma or less, while 2% were illiterate. Only 33% passed their 12th grade, out of which 12% of mothers had a graduate degree. Only 13% of mothers managed to hold down a job, and the majority of mothers (97%) remained homemakers. Seventy percent of their families earned between INR 5000 and INR 10,000 per month, with 23% and 7% of their families making less than INR 5000 and more than INR 100,000, respectively. Fifty-seven percent of mothers were from nuclear households, with mothers from joint families making up the remaining 43%. Seventy-seven percent of the mothers in our study sample were primiparous, while 23% were multiparous. Only 1% of mothers gave birth at home, while 99% of births took place in hospitals, 83% of which were handled by the government and 16% by the private sector. Seventy-one percent of deliveries were carried out normally vaginally, and 29% were caesarean sections. Of the babies delivered, 45% were female and 55% were male. Eighty-three percent of the babies were between six and 12 months old, and 17% were between 13 and 24 months old on average. The sociodemographic details of the study population are summarised in Table 1.

**Table 1:** Basic demographics of the study participants

Variables	Number	Percentage (%)
Age (in years)		
≤25	312	78
>25	88	22
Residence		
Rural	372	93
Urban	28	7
Education status		
No formal education	8	2
Grade 1 to Grade 4	63	15.8
Grade 5 to Grade 8	75	18.8
Grade 9 to Grade 10	122	30.5
Grade 11 to Grade 12	105	26.2
Grade 12 and above	27	6.8
Occupation		
Homemaker	348	87
Working	52	13
Family size		
Nuclear	332	83
Joint	68	17
Family income per month		
< $60	172	43
$60-$120	168	42
>$120	60	15
Parity		
Primi	308	77
≥ Para 2	92	23
Place of delivery		
Home	4	1
Institutional		
Government	332	83
Private	64	16
Mode of delivery		
Normal vaginal delivery	284	71
Caesarean section	116	29
Gender of the child		
Male	220	55
Female	180	45
Age of the child		
6 months to 12 months	324	81
12 months to 24 months	76	19

Knowledge on breastfeeding

Sixty-eight percent of mothers have heard the word "exclusive breastfeeding" (EBF). Only 9% of mothers learned this information through the media, whereas 29% and 62% of mothers said they learned it from family members and healthcare personnel, respectively. Unexpectedly, only 23% of mothers were aware that breastfeeding needs to be started as soon as the baby is born. With respect to the period of EBF, 45% of mothers said that only breast milk should be given until the child is six months old, while 28% of mothers were sceptical of the topic. Sixty-seven percent of mothers did not know what colostrum was. Thirty-nine percent of mothers said colostrum ought to be thrown away. Twenty percent of mothers were aware of prelacteal feeds, and honey was the most common prelacteal feed used. Fifty-three percent of mothers were able to express milk for their infants, compared to 47% who were unable to. Fifty-four percent of mothers were unsure if breastfeeding was good for the mother and the baby. Only 9% of mothers were aware of the duration of prolonged breastfeeding, while 57% were ignorant of the same. Table 2 lists the study subjects' understanding of breastfeeding.

**Table 2:** Study participants' knowledge of breastfeeding TIB: time to initiate breastfeeding; BF: breastfeeding

Questions and answers	Number	Percentage (%)
Have you heard of EBF?		
Yes	272	68
No	128	32
TIB after delivery		
Within 1 hour	92	23
After 1 hour	308	77
How long should EBF be continued?		
Less than 6 months	60	15
6 months	180	45
More than 6 months	48	12
Do not know	112	28
When should solid food be introduced?		
< 6 months	184	46
6 months	144	36
>6 months	72	18
Have you heard about colostrum?		
Yes	132	33
No	268	67
Do you think colostrum should be given to the baby or not?		
Should not be given	156	39
Should be given	244	61
Is colostrum good for the child?		
Yes	164	41
No	140	35
Do not know	96	24
Have you heard of prelacteal feeds?		
Yes	80	20
No	320	80
What are the names of some prelacteal feeds?		
Honey	64	16
Sweet	12	3
Others	4	1
Do you know how to express milk?		
Yes	212	53
No	188	47
Does BF have any health benefits both for the baby and the mother?		
Yes	116	29
No	68	17
Don’t know	216	54
How long should you continue BF?		
< 2 years	92	23
2 years	36	9
>2years	44	11
Don’t know	228	57
What are your sources of BF information?		
Family members	104	29
Healthcare professional	248	62
Media	36	9
Participant’s knowledge		
Good	196	49
Fair	144	36
Poor	60	15

Practice of breastfeeding

Thirty-six percent of the respondents had initiated breastfeeding within an hour, and 64% had done so later than that. Sixty-seven percent of infants received breast milk as their first feeding after birth, while 57% of mothers gave their newborns colostrum. In 33% of instances, prelacteal feed was administered. In 53% of the cases, breastfeeding was done exclusively, and 61% of the time, solid food was introduced at six months. After the age of six months, 70% of mothers fed their infants milk other than breast milk. The majority of women (81%) sustained breastfeeding their kids after the first six months. Table 3 shows the practices involved in breastfeeding.

**Table 3:** Responses on the practice of breastfeeding

Variables	Number	Percentages (%)
Breastfeeding was initiated within an hour of birth		
Yes	144	36
No	256	64
Colostrum was given to the baby		
Yes	228	57
No	172	43
The first feeding given to the baby was milk		
Yes	268	67
No	132	33
Prelacteal feeds were given to the baby		
Yes	132	33
No	268	67
EBF was given to the baby up to six months of age		
Yes	212	53
No	188	47
The baby was given other milk apart from breast milk after six months		
Yes	280	70
No	120	30
Solid food was introduced at six months of age		
Yes	244	61
No	156	39
Is breastfeeding still continued?		
Yes	324	81
No	76	19

A chi-square test was used to compare the awareness of the EBF with various socio-demographic variables. Working mothers, mothers with several children, mothers older than 25 years of age, and mothers with higher education levels showed a good understanding of EBF and came out to be statistically significant (p< 0.5). The monthly family income did not exhibit any statistically significant value (p> 0.5). The comparison is shown in Table 4.

**Table 4:** Comparison between sociodemographic characteristics and knowledge of EBF

Variables	Knowledge of EBF		p-value (chi-square test)
	Present	Absent	
Age of the mother (in years)			
≤25	164	148	0.001
˃25	64	24	
Education			
≤ Grade 10	128	140	0.000
> Grade 10	100	32	
Occupation			
Homemaker	192	164	0.000
Working mother	44	8	
Family income per month			
≤ $60	100	72	0.766
> $60	128	100	
Parity			
Primi	156	152	0.000
Multipara	72	20	

Another similar comparison was done between the socio-demographic variables and the practice of EBF itself using a chi-square test. Working mothers, mothers older than > 25 years of age, educational attainment (> 10th standard), and multiparous mothers who properly practised EBF were statistically significant (p< 0.5), although the monthly family income did not demonstrate any statistically significant value (p> 0.5). The comparison is shown in Table 5.

**Table 5:** Comparison between sociodemographic characteristics and practice of EBF

Variables	Practice of EBF		p-value (chi-square test)
	Present	Absent	
Age of the mother (in years)			
≤25	152	160	0.002
˃25	60	28	
Education			
≤ Grade 10	124	144	0.000
> Grade 10	88	44	
Occupation			
Housewife	202	146	0.000
Working mother	10	42	
Family income per month			
≤ $60	100	72	0.509
> $60	140	88	
Parity			
Primi	164	144	0.000
Multipara	72	20	

## Discussion

The objective of this study was to evaluate the degree of breastfeeding knowledge and practices among women seeking care for their sick child between the ages of six months and 24 months at Bankura Sammilani Medical College and Hospital, a rural tertiary healthcare centre in Bankura, the Indian state of West Bengal. In this district, the literacy rate for females (60.44%) is lower than the national average (65.46%) [7, 8]. Everybody is aware that female literacy plays a significant role in raising the success rate of any health programme, and breastfeeding programmes are not exempt from the list. Kerala is a remarkable exception, where U5 mortality is 13 per 1,000 live births, significantly lower than the national average of 45 per 1,000 live births [9]. A total of 400 mothers were included in the study, with 78% of them under the age of 25 and the remaining 22% above. Compared to the current study, Hasan M et al. found that 54.5% of mothers were under the age of 24 [10] since the current study included 77% of primi mothers whereas their study only included 52.9%. In accordance with the location of this medical college, 93% of mothers were from rural areas, while only 7% were from metropolitan areas. Only 33% of mothers had achieved the educational level of the 10+2 standard, while 67% had completed the tenth grade or less in terms of education.

Three crucial interventions-early breastfeeding initiation, exclusive breastfeeding for six months, and the prompt introduction of age-appropriate complementary foods-are crucial to achieving Millennium Development Goals (MDG) 1 and 4 and addressing child malnutrition and mortality, respectively [11, 12].

The results of the current study showed that the percentage of women who started breastfeeding within an hour of giving birth (36%) was lower than the corresponding national (41.8%) and state (59.85%) figures [13, 14], and far below the goal of 50% outlined in the Tenth Plan [15]. Breastfeeding began within an hour of the baby's birth for 36% of mothers, indicating a lack of information about the practice, which may have been caused by participants' low literacy levels and a lack of counselling at the time of antenatal visits. Studies have shown that India's rural areas have better access to healthcare than its neighbours. In contrast to the current study, Menon K et al. [16] reported that 84.1% of mothers practised exclusive breastfeeding in their study from a rural area of Thrissur District, Kerala, India. In addition, Menon K et al. observed that 15.8% of women provided prelacteal feeds, which is nearly twice as many as the present study's 33% finding. It's possible that the study population's poor literacy levels contribute to their ignorance of the advantages of breast milk as a newborn's only first feed. According to a study by Hossain M et al. [17] from rural Bangladesh, 35.9% of women exclusively breastfed their infants up to the age of 6 months. The current study found 53%, which is higher than the findings of Hossain M et al. but equivalent to a study by Bhattathiry MM et al. that found 60% [18]. In comparison to a study conducted by other researchers as well [19], the rate of EBF among illiterate mothers was low. Age >25 years, parity two or more, an educational level of grade 12 or higher, and being a housewife all positively affect EBF and are statistically significant (p-value 0.05); however, family income has no influence and is statistically insignificant (p-value 0.05). According to studies on mothers in India and Malaysia, housewives and mothers who are not employed are more likely to practice EBF than their counterparts [20, 21]. In the current study, 67% (n = 268) of mothers were unaware of the benefits of colostrum. Despite their inadequate knowledge, 61% of women refused to reject colostrum, which is comparable to studies by Wolde T et al. [22] and Tadele N et al. [23], both from Ethiopia, which revealed 63% and 60.29% of mothers, respectively, refused to discard colostrum. In the current study, 57% of mothers fed their infants colostrum as their first feeding. Contrary to the findings of the current study, Menon K et al. [16] found that 84.1% of breastfeeding mothers fed their babies breast milk as their first feeding. This discrepancy may be brought on by variations in the literacy rate, traditional beliefs and practices, the existing healthcare system, and, last but not least, perceptions of the significance of colostrum among the study populations of these two research projects.

Compared to research by Alamirew MW et al. [24] and Shirima R et al. [25], where 19.5% and 17.9% of mothers, respectively, agreed that prelacteal feeding is necessary for the baby, only 20% (n = 80) of mothers in the current study were aware of prelacteal feeding. In the current survey, 4% of mothers felt that due to its purgative effects, honey is the most popular prelacteal feed. Few people also think it affects the baby's future capacity to speak a more nuanced language. Despite being aware of the need for prelacteal feed, 33% of women supplied prelacteal feed to their infants as their first food, mimicking the findings of a prior study by Bhattathir MM, et al. [18], which indicated that 32% of mothers gave prelacteal food to their infants. Another study found that 19% of women engaged in prelacteal feeding [26]. It is obvious that prelacteal feeding practices differ regionally and culturally.

According to the current study, 45% of mothers are aware that EBF lasts for six months, which is equivalent to a study conducted by other authors that shows 44.1% are aware of it [17]. The highest knowledge of EBF (70.9%) was reported by Bayissa ZB et al. [27], and the lowest (23%) was recorded by Girard AM et al. [28]. Fifty-three percent of mothers in the current study exclusively breastfed their infants for the first six months, indicating a gap between study participants' awareness and actual EBF practice. Similar discrepancies were found by other researchers, who found that while 82.5% of nursing women understood the value of exclusive breastfeeding, only 44.1% were aware of how to practise EBF for up to six months [16].

The results of the current study showed that few respondents were aware of when to introduce solid food and how long to breastfeed babies. Only 36% of women are confident about the precise moment to introduce solid food, and only 9% are aware of the duration of breastfeeding. After the first six months, however, 61% of women started introducing complementary foods in addition to breast milk. It might be feasible because of social customs known as "Annaprasanna" or "MukheBhat" in the local tongue, which defend infants at risk of malnutrition brought on by the delayed introduction of solid food. According to a study by Cousens et al., there was a decrease in clinical malnutrition when prolonged breastfeeding was complemented by solid foods [29].

Only 29% of mothers knew the advantages of breastfeeding, according to the current study. As opposed to the current study, Bayissa ZB et al. and the Wana AD study showed that, respectively, 86.3% and 100% of mothers understood the importance of breastfeeding [27, 30]. This substantial disparity might be caused by variations in the study participants' prenatal visits, health advice acceptance rates, and education level. In contrast to family income, which had a p-value > 0.05 and was statistically insignificant, mothers who were older (> 25 years), had greater levels of education (> the 10th grade), were housewives, and had more children had a good understanding of and practice in breastfeeding. Except for family wealth, this result was consistent with the study conducted by Bangladeshi researchers Hassan M et al. [10]. However, Hassan M. et al. found that family income had a statistically significant p-value (<0.05), which is different from the results of the current study (p-value=>0.05) [10]

## Conclusions

This study found that levels of breastfeeding awareness and practice (53%) fell short of the national statistics (63.7% as per the National Family Health Survey 5 (NFHS)). and slightly higher than the WHO recommendations (50% by 2025). All helpful breastfeeding information should be disseminated in the community at large to improve the data currently available. Examples of effective methods include enhancing the current health care system, media promotion, and the addition of a required subject at the grade 12 level that covers the advantages of breastfeeding, the drawbacks of infant milk formula, and common health issues (i.e., acute gastroenteritis, acute respiratory tract infections, and so on). Millions of under-five children around the world will undoubtedly be protected if these strategies are employed.
